# A Giant Genome for a Giant Crayfish (*Cherax quadricarinatus*) With Insights Into *cox1* Pseudogenes in Decapod Genomes

**DOI:** 10.3389/fgene.2020.00201

**Published:** 2020-03-06

**Authors:** Mun Hua Tan, Han Ming Gan, Yin Peng Lee, Frederic Grandjean, Laurence J. Croft, Christopher M. Austin

**Affiliations:** ^1^Centre of Integrative Ecology, School of Life and Environmental Sciences Deakin University, Geelong, VIC, Australia; ^2^Deakin Genomics Centre, Deakin University, Geelong, VIC, Australia; ^3^Laboratoire Ecologie et Biologie des Interactions, UMR CNRS 7267 Equipe Ecologie Evolution Symbiose, Poitiers, France; ^4^School of Science, Monash University Malaysia, Petaling Jaya, Malaysia; ^5^Genomics Facility, Tropical Medicine and Biology Platform, Monash University Malaysia, Petaling Jaya, Malaysia

**Keywords:** freshwater crayfish, Parastacidae, genome, hybrid assembly, aquaculture, Illumina, Oxford Nanopore

## Introduction

The Decapoda represent a highly speciose and diverse group of crustaceans that provide important ecosystem services across mainly marine and freshwater environments, with many of the larger species being of significant commercial importance for fisheries and aquaculture industries. The value of generating and using genomic resources for key crustacean species and groups is widely appreciated for a variety of purposes including phylogenetic and population studies, selective breeding programs and broodstock management (Tan et al., 2016; Grandjean et al., 2017; Wolfe et al., 2019; Zenger et al., 2019; Zhang et al., 2019). However, comprehensive genomic studies are particularly limited for decapod crustaceans (Zenger et al., 2019; Zhang et al., 2019), compared to other aquatic invertebrate groups. Most genomic-related studies on decapod crustaceans have focused on mitogenome recovery and transcriptomics studies, with few genome assemblies attempted and most of these on commercially-important species (Song et al., 2016; Yuan et al., 2018; Zhang et al., 2019). Of the decapod species for which genome assemblies have been published and are publicly available, the quality is highly variable and BUSCO completeness poor with the exception of *Procambarus virginalis* (87.0%) and *Litopeneaus vannamei* (84.6%) (Table 1). It is now apparent that the assembly and annotation of decapod genomes are highly challenging due to the need to deal with large repetitive genomes, often with high heterozygosity, and should not to be undertaken by the faint-hearted or on a shoe-string budget (Zhang et al., 2019).

**Table 1:** Genomes used in this comparative study.

**Order**	**Sub/Infraorder**	**Species**	**Assembly size (bp)**	**Number of scaffolds**	**Scaffold N50 length (bp)**	**Longest scaffold (bp)**	**Sequencing technology**	**Source**	**References**
**Class Branchiopoda**									
Diplostraca	Anomopoda	*Daphnia pulex*	197,206,209	5,186	642,089	4,193,030	SS	Ensembl (V1.0)	Colbourne et al. (2011)
Diplostraca	N/A	*Eulimnadia texana*	120,535,642	108	18,070,303	42,684,797	PE, PB	NCBI (NKDA01)	Baldwin-Brown et al. (2017)
**Class Malacostraca**									
Amphipoda	Talitrida	*Parhyale hawaiensis*	2,752,560,740	278,189	20,228,728	75,825,039	PE, MP	NCBI (LQNS02)	Kao et al. (2016)
Decapoda	Astacidea	*Cherax quadricarinatus*	3,236,648,033	508,682	33,235	970,867	PE, ONT	NCBI (VSFE01)	This study
Decapoda	Astacidea	*Procambarus virginalis*	3,338,655,684	1,980,964	37,475	717,999	PE, LJD	NCBI (MRZY01)	Gutekunst et al. (2018)
Decapoda	Brachyura	*Eriocheir sinensis*	1,118,179,535	1,768,652	111,755	2,002,076	PE	GigaScience repo	Song et al. (2016)
Decapoda	Caridea	*Exopalaemon carinicauda*	6,699,723,695	9,470,451	962	135,963	PE	NCBI (QUOF01)	Li et al. (2019)
Decapoda	Caridea	*Neocaridina denticulata*	1,284,468,468	3,346,358	400	124,746	PE	requested from author	Kenny et al. (2014)
Decapoda	Dendrobranchiata	*Litopenaeus vannamei*	1,663,565,311	4,682	605,555	3,458,385	PE, PB, BAC	NCBI (QCYY01)	Zhang et al. (2019)
Decapoda	Dendrobranchiata	*Marsupenaeus japonicus*	1,660,270,162	2,434,740	912	29,048	PE	NCBI (NIUR01)	Yuan et al. (2018)
Decapoda	Dendrobranchiata	*Penaeus monodon*	1,447,415,504	2,525,346	769	26,545	PE	NCBI (NIUS01)	Yuan et al. (2018)

*Sequencing Technology abbreviations*.

*PE, Paired-end short reads (Illumina)*.

*MP, Mate-pair short reads (Illumina)*.

*LJD, Long jumping distance (Illumina)*.

*PB, Pacific Biosciences long reads*.

*ONT, Oxford Nanopore Technology long reads*.

*SS, Paired-end Sanger sequencing (plasmid, fosmid libraries)*.

The lack of genomic resources coupled with limited understanding of the molecular basis of gene expression and phenotypic variation will continue to limit advances in aquaculture-based productivity of decapods. Understanding the molecular basis of phenotypic variation and gene function is therefore important for selective breeding programs for traits such as increased growth and disease resistance (Zenger et al., 2019). Similarly, whole genome assemblies support genome-wide association studies (GWAS) to identify trait-specific loci and for genomic-based selective breeding.

Freshwater crayfishes make up an important group of Decapod crustaceans (Crandall and De Grave, 2017) naturally occurring on all continents, with the exception of Antarctica and Africa (Toon et al., 2010; Bracken-Grissom et al., 2014). They reach their greatest species richness in Australia and North America. A number of species are subject to aquaculture and recreational activities, indigenous fishers for food security and bait fisheries for anglers, and others are of conservation concern due to a range of activities threatening vulnerable freshwater environments (Piper, 2000; Richman et al., 2015; CABI, 2019). The genomic architecture and karyotype evolution of freshwater crayfish is also of interest as they have some of the largest chromosome numbers (*2n* = *200*) recorded for invertebrate species (Tan et al., 2004). In Australia, freshwater crayfish species from the genus *Cherax* Erichson, known as smooth yabbies, include the largest commercial crayfish species in the world (Austin, 1987, 1996; Austin and Knott, 1996). Of these, the economically-important and best known is the red claw crayfish, *Cherax quadricarinatus* von Martens, distributed widely across northern Australia (Austin, 1996; FAO, 2019). This species is capable of growing to greater than 200 grams making it the second largest commercial species of *Cherax* (Austin, 1987) behind the marron (*C. cainii* Austin) (Austin and Ryan, 2002). The popularity of the red claw as an aquaculture and ornamental species and its adaptability has resulted in widespread translocation, resulting in an extensive global distribution and it is acknowledged as a major invasive species of inland aquatic ecosystems in the tropics (Ahyong and Yeo, 2007; Larson and Olden, 2013; Saoud et al., 2013). Due to its large size and ease with which it can be maintained in captivity, *C. quadricarinatus* is also increasingly being used as a model organism to address fundamental questions relevant to molecular biology, physiology, functional genomics and cell biology (Fernández et al., 2012; Pamuru et al., 2012; Ventura et al., 2019).

The genetics of *C. quadricarinatus* has been well studied using PCR-based approaches and recently, next generation sequencing has been used for mitogenomic and transcriptomic studies (Baker et al., 2008; Gan et al., 2016; Tan et al., 2016). In this paper, we present the first genome assembly for a southern hemisphere crayfish using a hybrid assembly approach utilizing long Nanopore reads and short Illumina reads, which has proven to be an efficient and effective approach for the assembly and annotation of species with large and repetitive genomes (Austin et al., 2017; Tan et al., 2018; Gan et al., 2019; Sánchez-Herrero et al., 2019), but has only been minimally explored to support decapod assemblies (Van Quyen et al., 2020).

We then benchmark our assembly against seven other published decapod genome assemblies and present a preliminary phylogenomic analysis for decapod crustaceans. Lastly, we use these data to investigate the occurrence and diversification of nuclear mitochondrial DNA (NUMTs)^1^ in decapod genomes, which is directly relevant to debates on the veracity of the most common approach to the DNA barcoding of life for molecular-based taxonomic identification and biodiversity assessment based on the mitochondrial cytochrome oxidase 1 (*cox1*) gene region (Hebert et al., 2003; Cristescu, 2014; deWaard et al., 2018).

## Materials and Methods

### Data Generation

Tissue samples from two male *C. quadricarinatus* individuals were collected from Northern Territory in Australia, DWN1 from Rapid Creek and M2R2 from Charles Darwin University Aquaculture Centre, both located in Darwin following institutionally endorsed ethical, biosecurity and safety guidelines. Genomic DNA was extracted using E.Z.N.A.® Tissue DNA Kit (Omega Bio-tek) from tail muscle tissue. One microgram of DWN1 was sent to Macrogen, Korea for PCR-free library preparation and sequencing on the Illumina HiSeq platform. Additional Illumina sequencing on the Illumina NovaSeq 6000 system was also performed at the Deakin Genomics Centre using a PCR-based library constructed with NEBNext Ultra Illumina library preparation kit. Using tissue samples from DWN1 and M2R2 individuals, for each Nanopore library, ~1 μg of gDNA as measured by Qubit Fluorometer (Invitrogen) was processed using the LSK108 library preparation kit followed by sequencing on the R9.4 MinION flowcell. Nanopore data were base called with Albacore versions compatible with kits and flow cells used (discontinued, was available on https://community.nanoporetech.com) and adapter-trimmed with the Porechop tool (Wick, 2017).

### *De novo* Assembly and Scaffolding of the Crayfish Genome

Raw reads were pre-processed prior to assembly. The fastp tool (–*poly_g_min_len 1*) (Chen et al., 2018) was used to trim polyG sequences from the 3' end of reads generated on the Illumina NovaSeq platform. All short reads were trimmed with Trimmomatic (Bolger et al., 2014) to eliminate adapters (*ILLUMINACLIP:2:30:10*) and low quality sequences (*AVGQUAL:20*), and subsequently assembled with the Platanus assembler (Kajitani et al., 2014) using default parameters. The repeat content and large size of this genome necessitated scaffolding of the assembly with several data types; these included the use of short and long reads in order to span gaps of various sizes, in addition to using data from a previous *C. quadricarinatus* transcriptome project (Tan et al., 2016) to stitch together scaffolds containing coding genes. A first level of scaffolding was performed with Platanus, which scaffolds the assembled contigs and further closes gaps with insert size and sequence information from short paired-end libraries. Subsequently, as second level of scaffolding was accomplished with long Nanopore reads using LINKS (Warren et al., 2015), which was run for 20 iterations with multiple distance values (*d*) and step of sliding window (*t*), in addition to set values for k-mer (-*k 19*), minimum number of links (-*l 5*) and maximum link ratio between two best pairs (-*a 0.3*). Detailed parameters used in each iteration is available as Data Sheet 1. Resulting scaffolds were polished with pilon v1.22 (–*fix all*) (Walker et al., 2014) for five iterations. A third scaffolding step was performed using *C. quadricarinatus* RNA-seq reads previously generated (ERP004477) (Tan et al., 2016). HISAT2 was used to align RNA-seq reads to the assembly (Kim et al., 2019), information from which was used by BESST (Sahlin et al., 2014) for further scaffolding. All assemblies were evaluated for “completeness” based on the detection of single copy conserved genes (*arthropoda_odb9*) with BUSCO (Simão et al., 2015).

### Genome Size Estimation

Jellyfish (Marçais and Kingsford, 2011) was used to count the occurrence of 19-, 21- and 25-mers in pre-processed short reads, generating k-mer frequency histograms that were uploaded to the GenomeScope webserver (*max kmer coverage* disabled) (Vurture et al., 2017) for estimations of haploid genome size, heterozygosity, and repetitive content.

### Gene Prediction and Functional Annotation

A first iteration of the MAKER v2.31.10 annotation pipeline (Cantarel et al., 2008) was run to produce initial gene models based on transcript and protein hints (*est2genome* = *1*; *protein2genome* = *1*) from *C. quadricarinatus* transcriptome sequences (Tan et al., 2016), protein sequences from UniProt-SwissProt (Consortium, 2018) and an additional set of proteins from four other published decapod genomes: *Eriocheir sinensis* (Song et al., 2016), *Litopenaeus vannamei* (Zhang et al., 2019), *Marsupenaeus japonicas*, and *Penaeus monodon* (Yuan et al., 2018). These gene models were used to train two *ab initio* gene predictors, SNAP (Korf, 2004) and AUGUSTUS (Stanke et al., 2006), which were provided in a second MAKER iteration. Gene models resulting from this iteration were again used to retrain SNAP and AUGUSTUS before a third and final iteration of MAKER. Genes with Annotation Edit Distance values (AED) ≤ 0.5 were retained (Eilbeck et al., 2009). A small AED value points to a lesser degree of variance between the predicted gene and evidences/hints used in prediction. For functional annotation, DIAMOND (Buchfink et al., 2014) was used to find homology between the predicted genes and known proteins in the UniProtKB (Swiss-Prot and TrEMBL) database (Consortium, 2018). The predicted protein sequences were also scanned for motifs, signatures, and protein domains using InterProScan (Jones et al., 2014).

### Comparative Analyses of Published Decapod Genomes

Publicly-available genome assemblies for seven published decapod species (*Eriocheir sinensis, Exopalaemon carinicauda, Litopenaeus vannamei, Marsupenaeus japonicus, Neocaridina denticulata, Penaeus monodon, Procambarus virginalis*) (Kenny et al., 2014; Song et al., 2016; Gutekunst et al., 2018; Yuan et al., 2018; Li et al., 2019; Zhang et al., 2019) and three other non-decapod crustaceans (*Daphnia pulex, Eulimnadia texana, Parhyale hawaiensis*) (Colbourne et al., 2011; Kao et al., 2016; Baldwin-Brown et al., 2017) were downloaded (sources in Table 1) and compared in terms of genome and assembly sizes, repeat content and completeness. “Completeness” of a genome was assessed with BUSCO (Simão et al., 2015) based on the *arthropoda_odb9* databases. To obtain repeat profiles, repeat families were identified *de novo* using RepeatModeler (Smit and Hubley, 2019), which uses RepeatScout (Price et al., 2005) and RECON (Bao and Eddy, 2002) to scan for repeats. The output from this step contains a set of consensus sequences from each identified repeat family that was used by RepeatMasker (Smit et al., 2019) to mask repeats within the assembly.

### Preliminary Detection of NUMTs

The eight assembled genomes for *C. quadricarinatus, Er. sinensis, Ex. carinicauda, L. vannamei, M. japonicus, N. denticulata, Pe. monodon*, and *Pr. virginalis* were also scanned for the presence of putative NUMTs. The *blastn* tool (Altschul et al., 1990) was used to align the mitochondrial *cox1* gene of each species to its corresponding genome (exception: *Procambarus clarkii cox1* was used since the full *cox1* sequence for *Pr. virginalis* is unavailable on NCBI). Each output was filtered to eliminate alignments based on the following criteria:

1. Alignment length <100 nucleotides.
2. Alignment length that spans 95% of a genomic scaffold.
3. Alignment at the edge of a genomic scaffold.

An illustration of how these filters were applied is provided in Data Sheet 2. Sequences of these putative NUMTs were extracted based on alignment start and stop coordinates and were aligned with MAFFT (Katoh and Standley, 2013). IQ-TREE (Nguyen et al., 2014) was used to perform model testing and construct a maximum-likelihood (ML) phylogenetic tree (*-m TESTMERGE*).

### Phylogenetic Analyses

A ML tree was also constructed based on orthologous protein sequences predicted by BUSCO. This tree consists of six decapods (*C. quadricarinatus, Er. sinensis, L. vannamei, M. japonicus, Pe. monodon, Pr. virginalis*) with *D. pulex, Eu. texana* and *Pa. hawaiensis* as non-decapod outgroup species, rooted with *Drosophila melanogaster*. Since this analysis used sequences predicted by BUSCO, *N. denticulata* and *Ex. carinicauda* with low BUSCO completeness were excluded from this analysis. Briefly, protein sequences within each orthologous group (based on *metazoa_odb9* and *arthropoda_odb9*) were aligned with MAFFT (Katoh and Standley, 2013) and trimmed with Gblocks (Castresana, 2000). Only multiple sequence alignments containing sequences from all 10 species were retained and concatenated into a supermatrix with FASconCat-G (Kück and Longo, 2014). IQ-TREE (Nguyen et al., 2014) was used for initial model testing and construction of the ML tree (*-m TESTMERGE*), with support values indicated by the SH-like approximate likelihood ratio test (SH-aLRT) (Guindon et al., 2010) and ultrafast bootstrap (UFBoot) (Minh et al., 2013) (*-alrt 1000 -bb 1000*).

## Results and Discussion

### A Giant Genome for a Large Freshwater Crayfish

**Figure 1:**
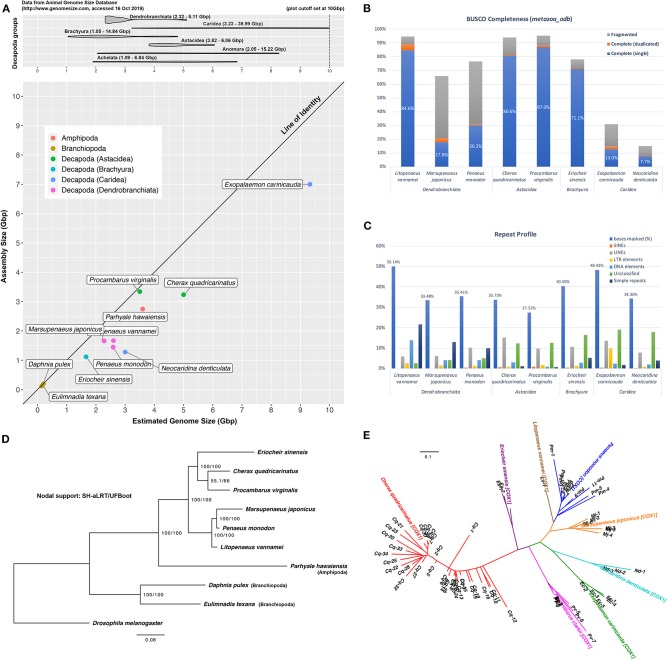
*Cherax quadricarinatus* and other published and publicly-available decapod genomes. **(A)** Top: range of genome sizes of decapod species in various sub- and infra-orders, based on information from the Animal Genome Size Database. Bottom: discrepancy between assembly and expected genome sizes of current available decapod genomes. **(B)** Genome “completeness” based on the *arthropoda_odb9* BUSCO dataset. **(C)** Masked repetitive regions of each genome and profiles of interspersed repeats. **(D)** Maximum-likelihood (ML) tree based on BUSCO predictions, rooted with *D. melanogaster*, with SH-aLRT/UFBoot values as indication of nodal support. **(E)** ML tree based on putative NUMTs identified from decapod genome assemblies.

This study produced 191 Gbp of HiSeq data (2 × 100 bp) and 774 Gbp of NovaSeq data (2 × 150 bp) in addition to 36 Gbp of Nanopore reads (average: 3,419 bp) to assist with scaffolding. Kmer-based methods estimated a 5 Gbp size for the *Cherax quadricarinatus* genome (Data Sheet 3), a value that is within the 3.82 to 6.06 Gbp size range that has been reported for species in the Infraorder Astacidea (Gregory, 2020) (Figure 1A). Based on this estimate, data generated in this study yields sequencing depths of 193× and 7× of short and long reads, respectively. Sequence data is available in the Sequence Read Archive (SRA) on NCBI (BioProject: PRJNA559771).

### Characteristics of the *C. quadricarinatus* Genome

Hybrid assembly of this genome resulted in a 3.24 Gbp final assembly contained in 508,682 scaffolds, with a N_50_ length of 33,235 bp (Table 1). Details of each assembly at each scaffolding level are available in Data Sheet 4. The BUSCO tool reports the presence of 81.3% and 12.8% of complete and fragmented arthropod BUSCOs, respectively. These values are comparable to the completeness evaluation of other recently sequenced decapod genomes that are relatively much smaller (*L. vannamei, Pr. virginalis, Er. sinensis*) (Table 1, Figure 1B). GenomeScope profiles display a small shoulder on k-mer distributions, indicating some level of heterozygosity within the genome and estimated an average heterozygosity level of 0.44%, which translates into approximately 1 mutation in 230 bp (Data Sheet 3). This is on par with the heterozygosity level of 0.53% reported for the marbled crayfish, *Pr. virginalis* (Gutekunst et al., 2018), and is higher than in prawns with both *M. japonicus* and *Pe. monodon*, both with 0.19% and 0.21% heterozygosity, respectively (Yuan et al., 2018). In addition, 33.73% of this assembly was masked for repeats, with a large proportion of interspersed repeats being long interspersed nuclear elements (LINEs), representing a repeat profile that is similar to that of *Pr. virginalis*, another crayfish species (Figure 1C). While the report of only 5.9% missing BUSCO genes in this *C. quadricarinatus* assembly suggests that majority of the gene space is present, the assembly reported in this study makes up only 64.8% of the expected 5 Gbp genome with 85.2% of short reads successfully mapped back to the assembly, indicating that there remains regions of this large genome that are missing from the assembly. This is not unusual for decapod genomes as they are rarely assembled to their full size, as seen from data points in Figure 1A, which mostly fall well below the “line of identity” between assembly vs. estimated genome size. The exception being *Pr. virginalis* with an assembled size close to its 3.5 Gbp genome size. This study tackles the ambitious assembly of a gigantic genome riddled with large repetitive structures and heterozygous regions that pose serious challenges to computational and bioinformatic resources, but there are clear benefits to hybrid assembly as an efficient method to add to the limited pool of genomic resources available for the order Decapoda. This assembly is available on NCBI (BioProject: PRJNA559771, WGS: VSFE00000000). It is noteworthy that this study has incorporated the largest dataset of long Nanopore reads to date for a decapod genome assembly (36 Gbp, ~7×), the other being the recent updated assembly of the black tiger prawn genome that incorporated the use of 2.5 Gbp of long Nanopore reads (~1.25×) (Van Quyen et al., 2020). The only other study to use long reads in decapod whole genome research is by Zhang et al. (2019) who utilized PacBio reads to support the assembly of the genome for the Pacific white shrimp *L. vannamei*.

### Predicted Genes and Functional

An average of 95.4% of RNA-seq reads from five *C. quadricarinatus* tissue types in Tan et al. (2016) were successfully aligned to the assembly. The annotation process predicted a total of 19,494 protein-coding genes, with an average gene length of 9,768 bp containing an average of 4.4 exons per gene. This number of predicted genes is within the range of that typically reported for other decapod crustaceans (*Er. sinensis*: 14436, *M. japonicus*: 16716, *Pe. monodon*: 18100, *Pr. virginalis*: 21772, *L. vannamei*: 25596). Of the annotated protein-coding genes, 88% are functionally annotated based on homology to existing UniProt protein sequences or through identification of protein domains and signatures. Protein and transcript sequences, BLAST homology alignments and InterProScan results are available as Data Sheet 5.

### Decapod Evolution Based on Nuclear Genes

Alignment and trimming of 97 orthologous genes resulted in a supermatrix of 16,679 amino acid characters. Maximum-likelihood analysis based on these protein sequences generated the tree shown in Figure 1D. Rooted with the fruit fly, the phylogeny recovers branchiopods and amphipods as outgroup species to the decapod species. Shrimp and prawn species (*L. vannamei, M. japonicus, Pe. monodon*) are clustered in a clade as the Dendrobranchiata, which in turn forms a sister relationship with a second clade consisting of the other three decapod species. In this latter clade, crayfish species are placed as sister taxa (*C. quadricarinatus* and *Pr. virginalis*), consistent with their taxonomic status as species within Astacidea, but with surprisingly weak nodal support (SH-aLRT 55.1%, UFBoot 66.0%). These relationships are consistent with findings reported in mitogenome- and nuclear-based studies (Bracken et al., 2009; Shen et al., 2013; Tan et al., 2019; Wolfe et al., 2019). Alignment and phylogenetic tree produced in this analysis are available as Data Sheet 6.

### Integration of the Mitochondrial *cox1* Gene into Decapod Genomes

The concept of DNA barcoding, by which a short universal DNA sequence is used to discriminate among species, is being very widely used to support taxonomic identification, detection of cryptic species and for establishing a reference database to support ecological, biodiversity and conservation-related studies (Ratnasingham and Hebert, 2007). A relatively recent and potentially powerful application of barcoding is the use of the rapidly increasing COI database as a reference for supporting environmental DNA metabarcoding studies (Deiner et al., 2017; Günther et al., 2018). However, DNA barcoding has its critics (Moritz and Cicero, 2004; Collins and Cruickshank, 2013), with a persistent concern being the impact of mitochondrial DNA copies in the nuclear genome (Bensasson et al., 2001; Hazkani-Covo et al., 2010) on the veracity of the DNA barcoding methodology, potentially making DNA barcoding unreliable for certain taxonomic groups (Sorenson and Quinn, 1998), including crustaceans (Song et al., 2008).

NUMTs have now been reported in a diversity of animal groups including crustaceans, with cytochrome oxidase 1 (*cox1*) NUMTs found in planktonic copepods (Bucklin et al., 1999) and snapping shrimp (Williams and Knowlton, 2001). Nguyen et al. (2002) and Munasinghe et al. (2003) reported the first NUMTs for crayfish, from the genus *Cherax*, and Song et al. (2008) for species of *Orconectes*. Our study extends the findings of Song et al. (2008) in that we identify *cox1* pseudogenes in each of the decapod genome assemblies we have examined, but find the number to be widely variable among taxa. This analysis identified the most *cox1* pseudogenes in *C. quadricarinatus* (34 insertion sites), followed by *Pe. monodon* (11), *Pr. virginalis* (8), *M. japonicus* (6), *Ex. carinicauda* (5), *N. denticulata* (3), *Er. sinensis* (2), and *L. vannamei* (1) (Figure 1E). NUMTs identified from these decapod species form monophyletic groups by species in the phylogeny, suggesting that the *cox1* mitochondrial gene was integrated into each nuclear genome subsequent to the divergence of species in this analysis, with either multiple independent transfer events or further evolution of NUMTs within each species through duplication events. The NUMT phylogenetic tree is available in Data Sheet 6.

In a broad-based study of NUMTs in 85 eukaryotic genomes, Hazkani-Covo et al. (2010) found a correlation with genome size. While finding the highest number of NUMTs in the red claw crayfish, with the second largest decapod genome assembled, is consistent with this observation, the evidence for a similar overall trend is not so clear across all the decapod species. In this context, it should be noted that we only have a relatively small sampling of decapod genomes and the varying assembly quality and volume of sequence data among studies makes testing this hypothesis fraught at this stage. There are also several caveats associated with this analysis. The identification process is dependent on the search methods and implemented filters, which if too conservative, can limit detection and exclude true NUMTs with highly divergent and variable sequences, especially if the time of insertion from the mitogenome occurred millions of years ago (Tsuji et al., 2012). It is also unknown whether other published decapod studies have been post-processed to exclude scaffolds containing mitochondrial genes during the submission process to databases. Nevertheless, results from this analysis provide an insight into the prevalence of NUMTs in decapod genomes, encouraging further exploration into the evolution of NUMTs in future studies.

## Conclusion

Following on from mitogenomic and transcriptomic studies, we present the first draft genome for the red claw crayfish (*Cherax quadricarinatus*) based on relatively large volumes of short and long genomic reads from Illumina and Oxford Nanopore (ONT) platforms. While the assembly is relatively fragmented, it is much better than many of those currently available for decapod species, and the quality of the annotation is equivalent to other more recently-sequenced decapod genomes. Due to the very large size and repetitive structures of the red claw genome, the assembly was highly challenging. However, we demonstrated the value of long Nanopore reads and a hybrid assembly approach for improving an assembly based on short reads alone (Data Sheet 4), which gives encouragement for tackling other crayfish and crustacean taxa with large and complex genomes. This draft genome will be an important and valuable resource to support ongoing comparative genomic, phylogenomics and molecular-based breeding studies for aquaculture, conservation and biodiversity-related studies and can be approved upon over time, with the generation of additional long read data. However, a major challenge still remains in relation to the computational resources needed to assemble large repetitive genomes from predominately short reads, even when aided with long reads (Lewin et al., 2018). Computationally, assembly, scaffolding and polishing processes to achieve the final draft genome took almost 85 processor-weeks, and annotation required another 100 processor-weeks. A worthwhile next step would be to investigate the efficacy of a long-read led crayfish genome assembly, now feasible as result of declining costs and improved accuracy of long read sequencing, and which should be less expensive and lead to greatly reduced processor time for assembly tasks.

## Data Availability Statement

The datasets generated for this study can be found in the NCBI BioProject PRJNA559771, BioSamples SAMN12558911 and SAMN12885547 (WGS accession: VSFE00000000; SRA accessions: SRR10214688, SRR10416208, SRR10416210, SRR10445782, SRR10445783, SRR10445784, SRR10484712).

## Author Contributions

CA, HG, and LC conceived the project. CA collected the samples. HG and YL performed the sequencing. MT analyzed the data. MT and CA wrote the manuscript. FG and LC modified the manuscript. All authors read and approved the final version of the manuscript.

### Conflict of Interest

The authors declare that the research was conducted in the absence of any commercial or financial relationships that could be construed as a potential conflict of interest.

## Footnotes

^1^The natural integration of DNA from mitochondria into the nuclear genome resulting in nuclear copies of mitochondrial pseudogenes (NUMTs).
