# Supplementary material for: A Giant Genome for a Giant Crayfish (Cherax quadricarinatus) With Insights Into cox1 Pseudogenes in Decapod Genomes
Source: Front Genet. 2020 Mar 6;11:201. doi: 10.3389/fgene.2020.00201 (PMC7069360; doi:10.3389/fgene.2020.00201)
Supplement: Supplementary file 1 [file Data_Sheet_1.PDF]

Supplementary Data: Parameters used for each iteration in scaffolding with LINKS v1.8.5 (Warren et al., 2015).

Iteration	d	t	k	l	a
1	1,000	50	19	5	0.3
2	2,500	25	19	5	0.3
3	5,000	10	19	5	0.3
4	7,500	5	19	5	0.3
5	10,000	5	19	5	0.3
6	15,000	5	19	5	0.3
7	20,000	2	19	5	0.3
8	25,000	2	19	5	0.3
9	30,000	2	19	5	0.3
10	35,000	2	19	5	0.3
11	40,000	2	19	5	0.3
12	45,000	2	19	5	0.3
13	50,000	2	19	5	0.3
14	55,000	2	19	5	0.3
15	60,000	2	19	5	0.3
16	65,000	2	19	5	0.3
17	70,000	2	19	5	0.3
18	75,000	2	19	5	0.3
19	80,000	2	19	5	0.3
20	85,000	2	19	5	0.3

d : *distance between k-mer pairs (ie. target distances to re-scaffold on)*
t : *step of sliding window when extracting k-mer pairs from long reads*
k : *k-mer value*
l : *minimum number of links (k-mer pairs) to compute scaffold (default -l 5)*
a : *maximum link ratio between two best contig pairs (default -a 0.3)*

References

Warren, R.L., Yang, C., Vandervalk, B.P., Behsaz, B., Lagman, A., Jones, S.J.M., et al. (2015). LINKS: Scalable, alignment-free scaffolding of draft genomes with long reads. *GigaScience* 4(1), 35. doi: 10.1186/s13742-015-0076-3.
