# Supplementary material for: A Giant Genome for a Giant Crayfish (Cherax quadricarinatus) With Insights Into cox1 Pseudogenes in Decapod Genomes
Source: Front Genet. 2020 Mar 6;11:201. doi: 10.3389/fgene.2020.00201 (PMC7069360; doi:10.3389/fgene.2020.00201)
Supplement: Supplementary file 2 [file Data_Sheet_2.pdf]

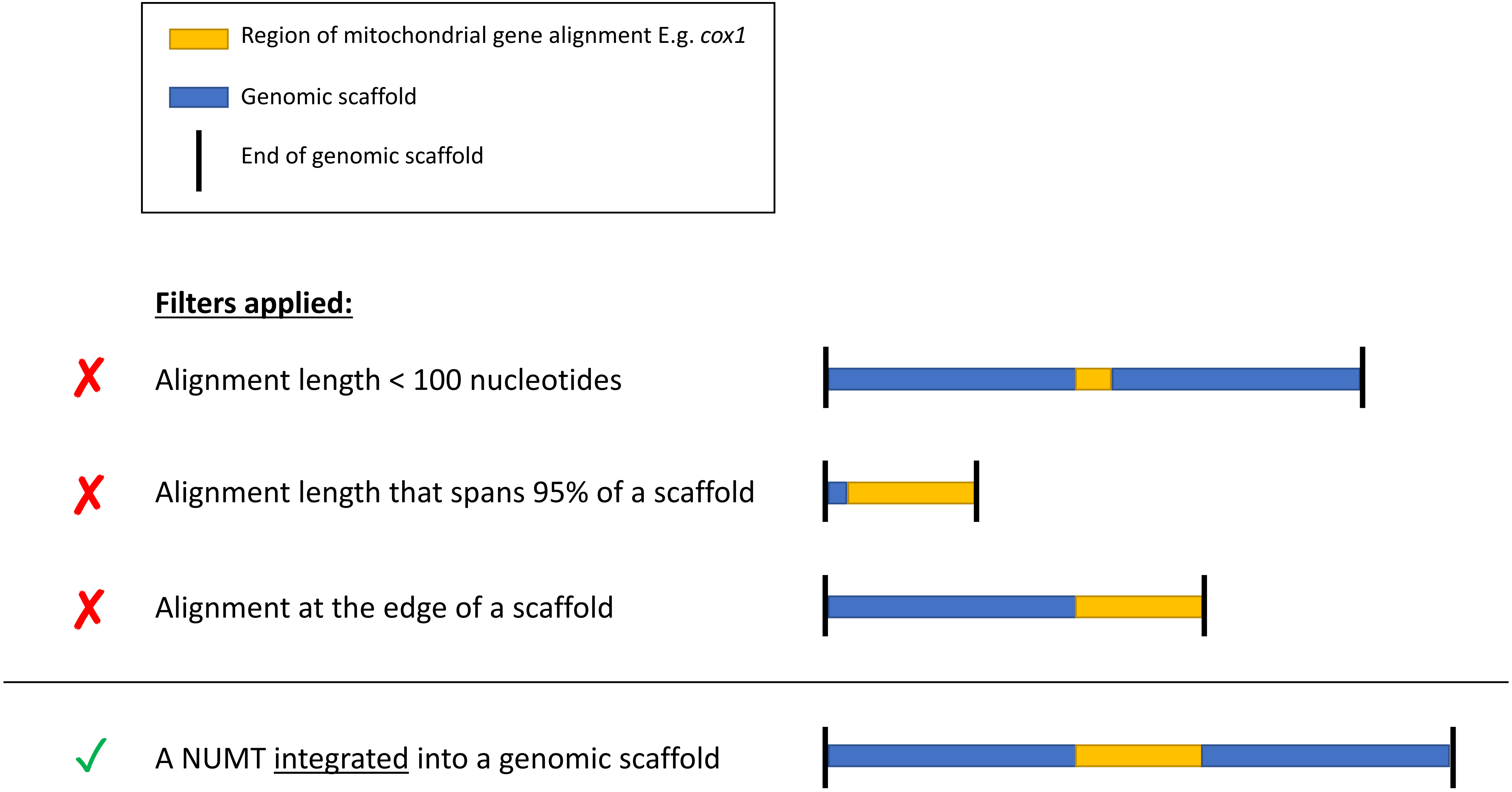
Region of mitochondrial gene alignment E.g. *cox1*
Genomic scaffold
End of genomic scaffold
Filters applied:
✗ Alignment length < 100 nucleotides
✗ Alignment length that spans 95% of a scaffold
✗ Alignment at the edge of a scaffold
✓ A NUMT integrated into a genomic scaffold
