# Supplementary material for: A Giant Genome for a Giant Crayfish (Cherax quadricarinatus) With Insights Into cox1 Pseudogenes in Decapod Genomes
Source: Front Genet. 2020 Mar 6;11:201. doi: 10.3389/fgene.2020.00201 (PMC7069360; doi:10.3389/fgene.2020.00201)
Supplement: Supplementary file 3 [file Data_Sheet_3.pdf]

Supplementary Data: GenomeScope profiles for reads based on k-mers 19, 21 and 25.

k = 19

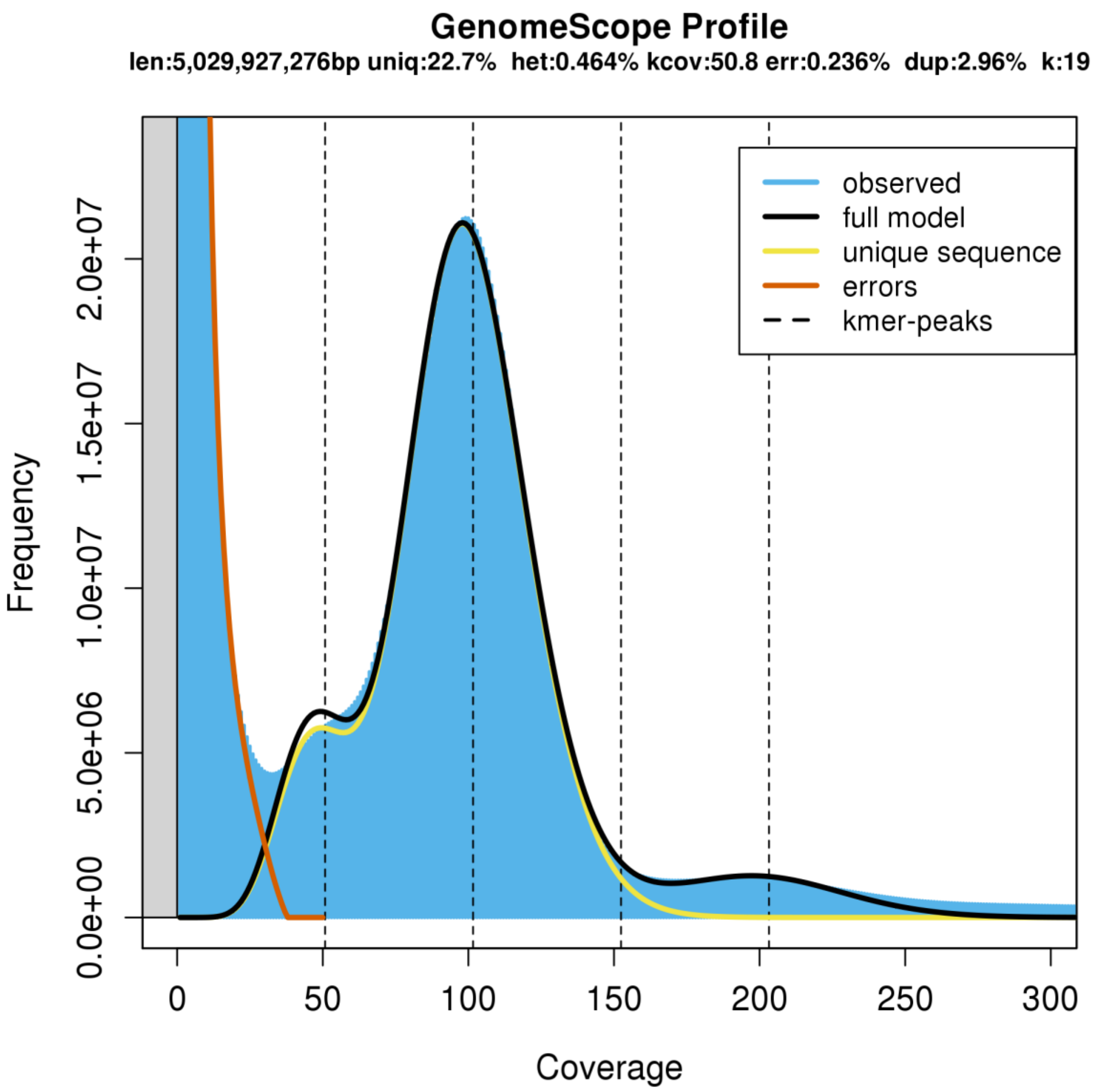

k = 21

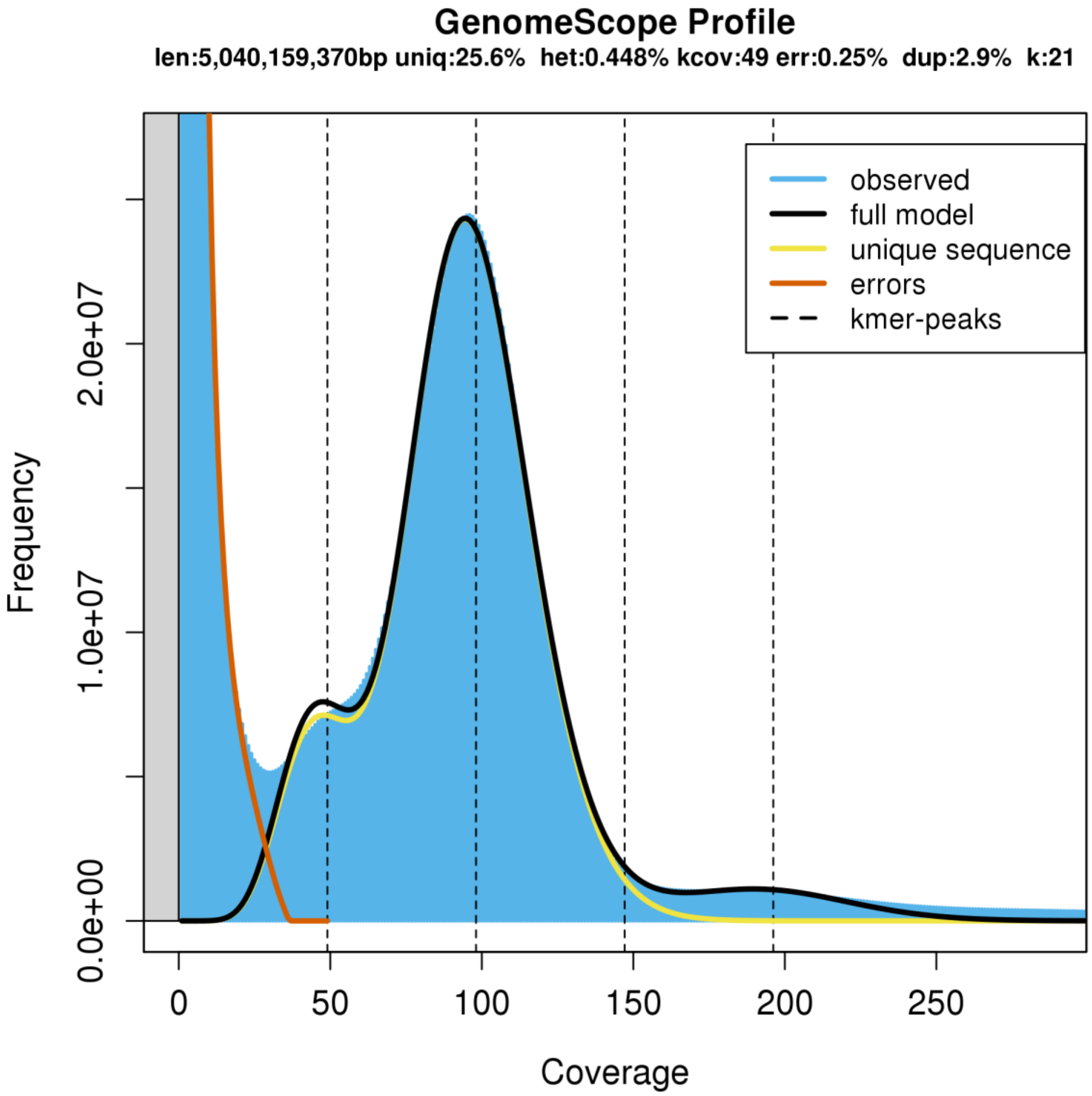

GenomeScope Profile
len:5,040,159,370bp uniq:25.6% het:0.448% kcov:49 err:0.25% dup:2.9% k:21
observed
full model
unique sequence
errors
kmer-peaks
Frequency
0.0e+00
1.0e+07
2.0e+07
0
50
100
150
200
250
Coverage

k = 25

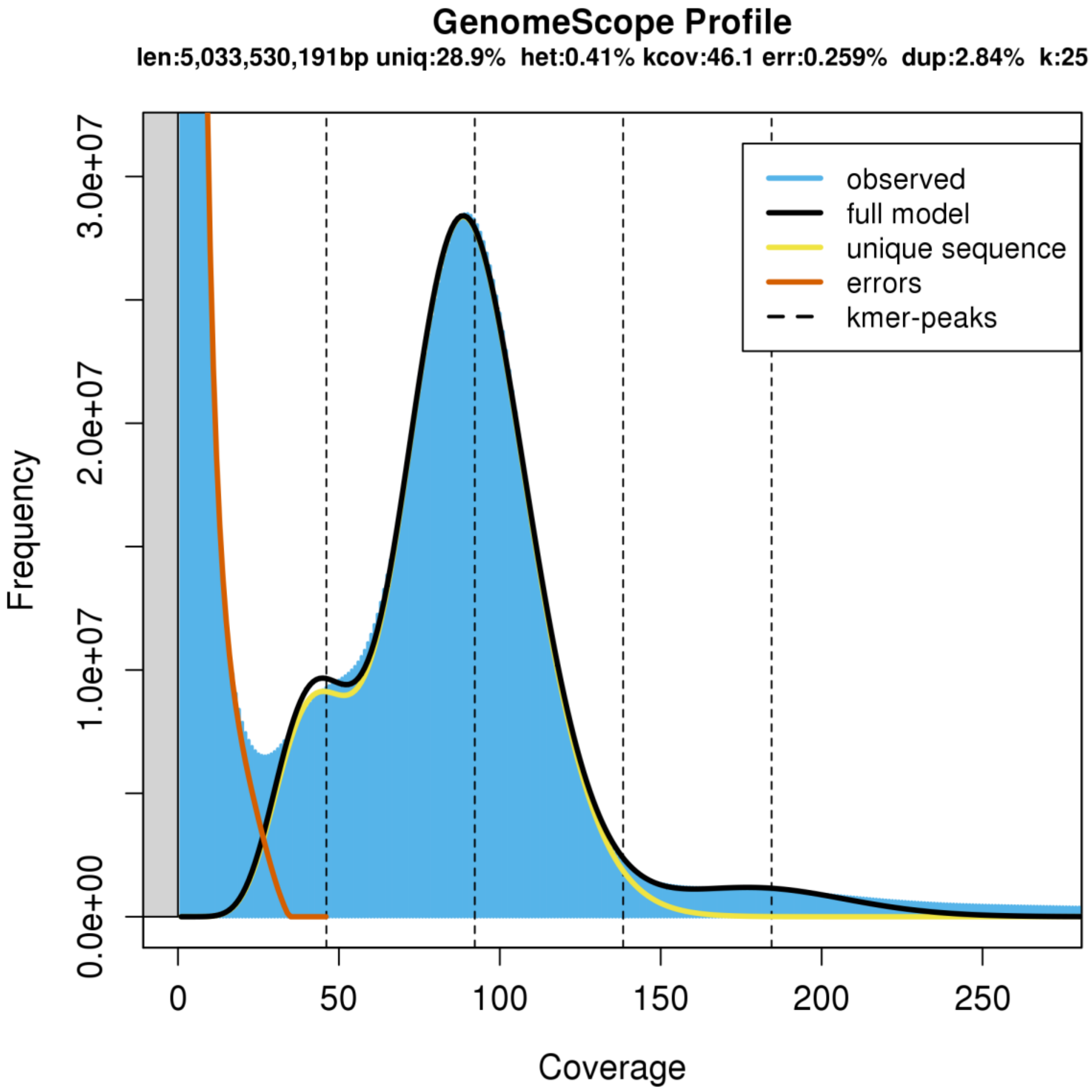

GenomeScope Profile
len:5,033,530,191bp uniq:28.9% het:0.41% kcov:46.1 err:0.259% dup:2.84% k:25
observed
full model
unique sequence
errors
kmer-peaks
Frequency
0.0e+00
1.0e+07
2.0e+07
3.0e+07
0
50
100
150
200
250
Coverage
