# Supplementary material for: A Giant Genome for a Giant Crayfish (Cherax quadricarinatus) With Insights Into cox1 Pseudogenes in Decapod Genomes
Source: Front Genet. 2020 Mar 6;11:201. doi: 10.3389/fgene.2020.00201 (PMC7069360; doi:10.3389/fgene.2020.00201)
Supplement: Supplementary file 4 [file Data_Sheet_4.pdf]

Summary of sequencing, assembly (with various levels of scaffolding) and annotation of the *Cherax quadricarinatus* genome

	**After Platanus (contigs)**	**After Platanus (scaffolds)**	**After LINKS (scaffolds)**	**After BESST (scaffolds)**
**Genome size estimation**				
Average based on 19-, 21-, 25-mers		5.03 Gbp		
**Genome assembly**				
Assembly size (bp)	5,722,527,322	2,975,290,328	3,235,773,168	3,236,648,033
Number of sequences	23,946,419	4,981,723	519,929	508,682
Sequence N50 length (bp)	245	1,403	30,510	33,235
Average sequence length (bp)	238.97	597.24	6,223.49	6,362.81
Longest sequence (bp)	423,127	423,007	970,867	970,867
Shortest sequence (bp)	124	200	206	500
**Genome completeness (*arthropoda_odb9*) (%)**				
Complete BUSCOs	17.0	28.2	68.6	81.3
Complete and single BUSCOs	16.3	27.4	67.8	80.6
Complete and duplicated BUSCOs	0.7	0.8	0.8	0.7
Fragmented BUSCOs	27.7	36.4	21.4	12.8
Missing BUSCOs	55.3	35.4	10.0	5.9
**Genome annotation**				
Number of predicted PCGs	-	-	-	19,494
Number of annotated PCGs	-	-	-	17,182
- Hits to UniProtKB	-	-	-	14,656
- Hits to protein domains	-	-	-	15,153
Average length of PCGs	-	-	-	353 aa
Longest PCG	-	-	-	8,231 aa (*I-connectin*)
